# Combined inhibition of glycolysis, the pentose cycle, and thioredoxin metabolism selectively increases cytotoxicity and oxidative stress in human breast and prostate cancer

**DOI:** 10.1016/j.redox.2014.12.001

**Published:** 2014-12-10

**Authors:** Ling Li, Melissa A. Fath, Peter M. Scarbrough, Walter H. Watson, Douglas R. Spitz

**Affiliations:** a Department of Radiation Oncology, Free Radical and Radiation Biology Program, Holden Comprehensive Cancer Center, University of Iowa, Iowa City, IA 52242, USA; b Duke Cancer Institute, Duke University, Durham, NC 27705, USA; c Division of Gastroenterology, Hepatology and Nutrition, Department of Medicine, University of Louisville, Louisville, KY 40292, USA

**Keywords:** 2DG, 2-deoxy-d-glucose, NAC, *N*-acetylcysteine, GSH, glutathione, GSSG, glutathione disulfide, DHEA, dehydroepiandrosterone, Au, auranofin, G6PDH, glucose-6-dehydrogenase, ROS, reactive oxygen species, Trx, thioredoxin, TrxR, thioredoxin reductase, Dehydroepiandrosterone, Pentose phosphate pathway, Oxidative stress, Auranofin, Buthionine sulfoximine, Glutathione, Thioredoxin, 2-Deoxy-d-glucose, Cancer

## Abstract

Inhibition of glycolysis using 2-deoxy-d-glucose (2DG, 20 mM, 24–48 h) combined with inhibition of the pentose cycle using dehydroepiandrosterone (DHEA, 300 µM, 24–48 h) increased clonogenic cell killing in both human prostate (PC-3 and DU145) and human breast (MDA-MB231) cancer cells *via* a mechanism involving thiol-mediated oxidative stress. Surprisingly, when 2DG+DHEA treatment was combined with an inhibitor of glutathione (GSH) synthesis (l-buthionine sulfoximine; BSO, 1 mM) that depleted GSH>90% of control, no further increase in cell killing was observed during 48 h exposures. In contrast, when an inhibitor of thioredoxin reductase (TrxR) activity (Auranofin; Au, 1 µM), was combined with 2DG+DHEA or DHEA-alone for 24 h, clonogenic cell killing was significantly increased in all three human cancer cell lines. Furthermore, enhanced clonogenic cell killing seen with the combination of DHEA+Au was nearly completely inhibited using the thiol antioxidant, N-acetylcysteine (NAC, 20 mM). Redox Western blot analysis of PC-3 cells also supported the conclusion that thioredoxin-1 (Trx-1) oxidation was enhanced by treatment DHEA+Au and inhibited by NAC. Importantly, normal human mammary epithelial cells (HMEC) were not as sensitive to 2DG, DHEA, and Au combinations as their cancer cell counterparts (MDA-MB-231). Overall, these results support the hypothesis that inhibition of glycolysis and pentose cycle activity, combined with inhibition of Trx metabolism, may provide a promising strategy for selectively sensitizing human cancer cells to oxidative stress-induced cell killing.

## Highlights

• Inhibition of both glycolysis and pentose cycle causes oxidative stress in human breast and prostate cancer cells.
• Combining inhibition of glycolysis and pentose cycle with inhibition of thioredoxin reductase enhances cell killing of these human cancer cells.
• The toxicity and oxidative stress is selective for cancer vs. normal cells.

## Graphical abstract

**Figure:**
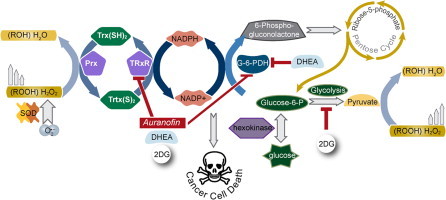

## Introduction

**Fig. 1:**
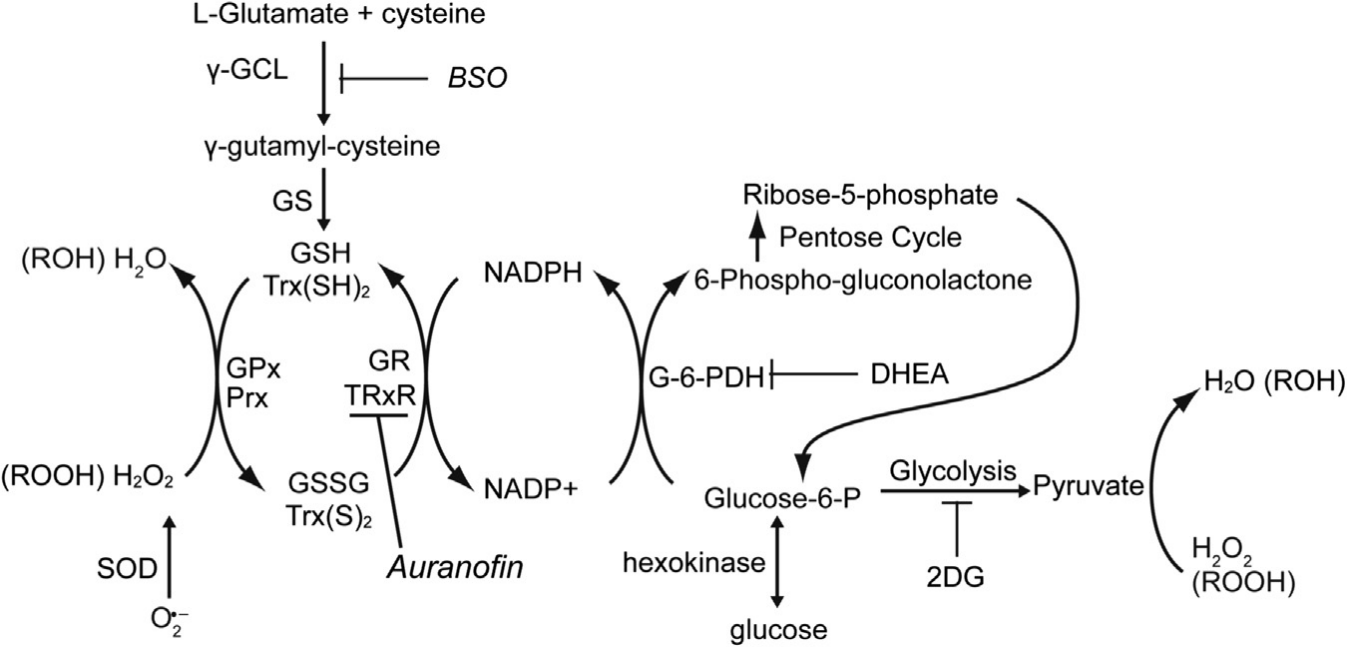
The pathways involving glucose and hydroperoxide metabolism believed to be involved with protection of cancer cells from metabolic oxidative stress (inhibitors of Trx and GSH metabolism are shown in italics). 2DG competes with glucose for uptake into the cells competitively inhibiting pyruvate production and the pentose cycle after glucose-6-phosphate-dehydrogenase (G6PD). DHEA inhibits G6PD. The GSH and Trx dependent systems participate in the detoxification of H_2_O_2_ and organic hydroperoxides. NADPH is a source of reducing equivalents for the Trx/GSH-dependent systems. BSO inhibits glutamate cysteine ligase (γ-GCL) preventing glutathione synthesis. Auranofin is the inhibitor of thioredoxin reductase (TrxR), which reduces the oxidized Trx to the reduced form. These inhibitors were used alone and in combination to increase the cancer cell oxidative stress, resulting in cancer cell cytotoxicity.

Cancer cells, relative to normal cells, demonstrate up regulation of glucose metabolism and a loss of regulation between glycolysis and aerobic respiration [1], [2], [3]. Growing evidence supports the hypothesis that tumor cells have altered mitochondrial metabolism leading to increased steady-state levels of intracellular reactive oxygen species (ROS) including superoxide (O_2_^•−^) and hydrogen peroxide (H_2_O_2_) [4], [5], [6], [7], [8], [9], [10]. It has also been hypothesized that cancer cells compensate for increases in steady-state levels of ROS by increasing glycolysis and pentose cycle activity to provide reducing equivalents for hydroperoxide metabolism (Fig. 1) [4], [5], [6], [7], [8], [9]. Glucose provides electrons for hydroperoxide metabolism *via* the activity of the pentose cycle to regenerate nicotinamide adenine dinucleotide phosphate (NADPH) to serve as the electron donor for glutathione (GSH) and thioredoxin (Trx) dependent peroxidase activity as well as through glycolysis to form pyruvate that can directly react to detoxify hydroperoxides through a decarboxylation reaction (Fig. 1) [8], [11], [12].

Consistent with the hypothesis that cancer cells have increased glycolysis and pentose cycle activity as a mechanism of protection against increased fluxes of hydroperoxides, inhibition of these pathways through glucose deprivation is known to cause selective oxidative stress and cytotoxicity in cancer cells versus normal cells [9], [13], [14]. The glucose analog, 2-deoxyglucose, inhibits glycolysis and cannot be fully oxidized in the pentose cycle, regenerating only half as much NADPH as a molecule of glucose [15]. Previous studies have demonstrated that 2DG treatment disrupts the NADP^+^/NADPH balance [16], [17], is cytotoxic to tumor cells *in vitro*[18], [19] and enhances the inhibition of tumor growth by agents that kill cancer cells via an oxidative stress mechanism *in vivo* [16], [20], [21].

Glucose-6-phosphate dehydrogenase (G6PDH) is the rate limiting enzyme in the oxidation of glucose through the pentose phosphate pathway. G6PDH catalyzes the chemical reaction of d-glucose-6-phosphate to 6-phospho-d-glucono-lactone regenerating NADPH (Fig. 1) [22]. Studies have shown that G6PDH expression and activity is increased in tumor tissues compared with normal cells [23], [24] and is strongly related to cellular oxidative stress responses [25]. Dehydroepiandrosterone (DHEA) is an endogenous primate steroid precursor that has been shown to be an inhibitor of mammalian G6PDH [26], [27]. It has been shown that treatment with DHEA leads to a 30–40% decrease of NADPH/NADP^+^ ratio, which may compromise cellular hydroperoxide metabolism [26].

GSH and Trx are cellular thiol redox cofactors that participate in redox sensitive signaling pathways, scavenging hydroperoxides and allowing for the maintenance of cellular redox potential. Studies have demonstrated that these antioxidant systems are up regulated in multiple cancer types compared to matched non-cancerous tissue [28], [29], [30], [31], [32]. In this regard up-regulation of GSH and Trx metabolism in breast and prostate cancer is correlated with disease progression and poor patient outcomes [29], [32]. The rate limiting step in GSH synthesis is glutamate cysteine ligase, which is inhibited by buthionine sulfoximine (Fig. 1; BSO). Trx is maintained in the reduced state by thioredoxin reductase (Fig. 1; TrxR). Auranofin (Au; Fig. 1) is a potent inhibitor of both cytosolic and mitochondrial TrxR [33], [34]. We have previously demonstrated that simultaneous inhibition of the GSH and Trx pathways results in cancer cell death via metabolic oxidative stress [35], [36], [37].

To determine drug combinations that were less toxic to normal versus cancerous human cells that could selectively cause metabolic oxidative stress induced cell-killing in cancer cells, the current study focused on combining pharmacological agents that inhibit glycolysis and the pentose cycle (2-DG and DHEA) with inhibitors of thiol-dependent hydroperoxide metabolism (BSO and Au). Treatment of human prostate and breast cancer cells with either 2DG or DHEA was found to decrease clonogenic cell survival and cell killing was further enhanced by combining both agents. Although this decrease in cancer cell survival was associated with disruptions in GSH metabolism, depleting GSH using BSO did not further enhance clonogenic cell killing. In contrast, inhibiting Trx metabolism using Au resulted in significantly increased clonogenic cell death when combined with DHEA or 2DG+DHEA that was reversed using NAC a small molecule thiol antioxidant. Importantly, normal human mammary epithelial cells (HMEC) were not as sensitive to 2DG, DHEA, and Au induced cell killing as their cancer cell counterparts (MDA-MB-231). These results support the hypothesis that cancer cells are more dependent on glucose as well as hydroperoxide metabolism than are normal cells and that combining inhibitors of glycolysis and the pentose cycle with Au may represent a promising approach for selectively causing oxidative stress-induced cell killing in breast and prostate cancer cells.

## Results

### DHEA inhibits G-6-PDH activity and enhances 2DG cell killing in breast and prostate cancer cells

**Fig. 2:**
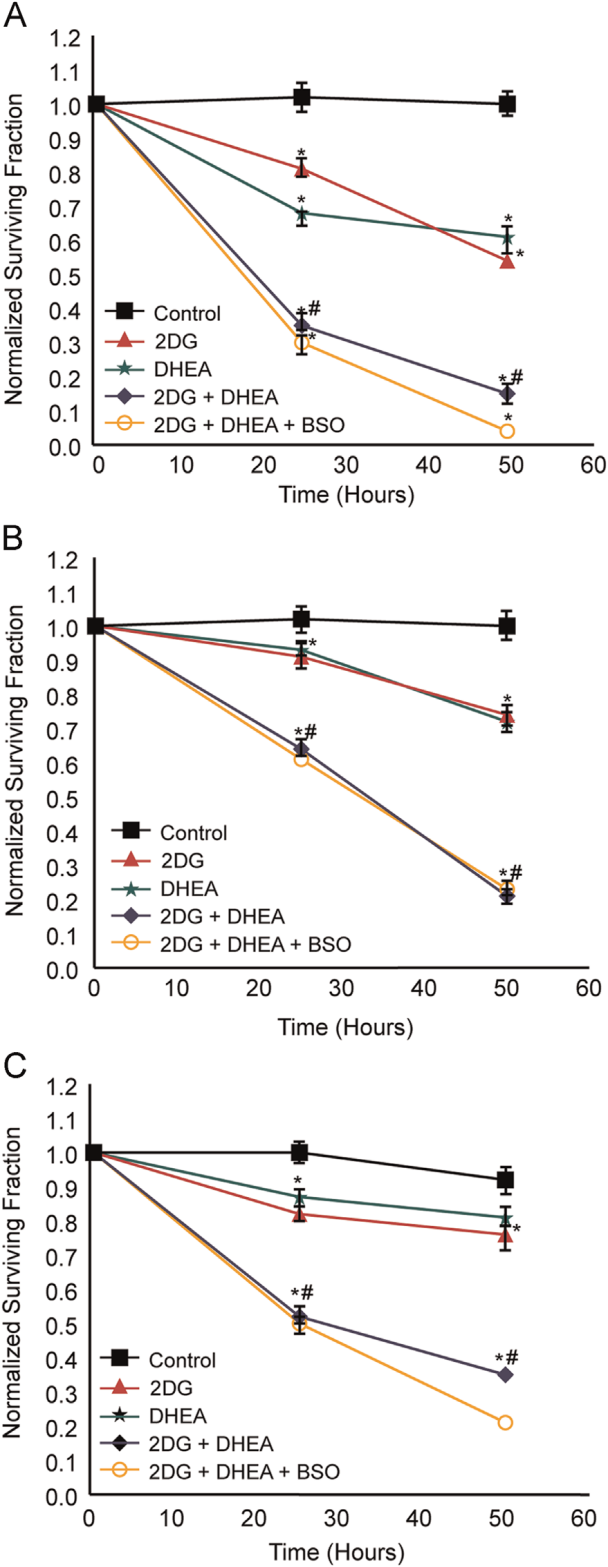
Clonogenic cell survival curves for PC-3 cells (A), DU145 cells (B), and MDA-MB-231 cells (C) treated with 2DG, DHEA, and BSO. 500,000–1,000,000 Cells were plated in 60 mm dishes. After 24 h cells were treated with 20 mM 2DG, 300 µM DHEA, and 1 mM BSO. Cells were collected for clonogenic survival assay at the 24, and 48 h after the treatment started. Each measurement represents mean±1 SD from two experiments. *p<0.001 difference versus control treatment, ^#^*p*<0.001 difference versus 2DG or DHEA treatment alone. One-way ANOVA was used with Tukey's post-hoc analysis was used to test for statistical significance.

We have previously determined that 2DG inhibits cancer cell growth through an oxidative stress mechanism in multiple cancer cell lines including MDA-MB231 breast cancer cells [20], [35], [37], [38], [39]. To test the hypothesis that an inhibitor of G6PDH could further enhance metabolic oxidative stress caused by 2DG (Fig. 1) MDA-MB231 breast cancer cells, PC-3 and DU145 prostate cancer cells were treated with 2DG and/or DHEA for 24 and 48 h followed by clonogenic cell survival assay (Fig. 2). DHEA inhibits the activity of human recombinant G6PDH with an IC50 of ~330 µM *in vitro* [27]. In the current studies 300 µM DHEA significantly inhibited G6PDH activity 35–50%, in all three of the cancer cell lines (Table 1). 20 mM 2DG was used to ensure that a relevant ratio of 2DG to glucose (≈1.8) was used to competitively inhibit glucose metabolism in the cells grown in RPMI 1640 medium, which contains 11 mM glucose. As expected, treatment with 2DG or DHEA decreased surviving fractions of all 3 cell lines by 10–20% or 20–40% after 24 or 48 h, respectively (Fig. 2A–C). Interestingly, treating the cells with DHEA in combination with 2DG significantly inhibited clonogenic cell survival compared to treatment with 2DG or DHEA alone, at both 24 and 48 h, in all three cancer cell lines tested (Fig. 2A–C). These results support the hypothesis that simultaneous disruption of glucose metabolism using both a glycolysis inhibitor (2DG) and an inhibitor of the pentose cycle (DHEA) enhanced cancer cell killing.

**Table 1:** G-6-PDH activity (mU/mg) on the PC-3, DU145, and MD-MB231 cells treated with 300 µM DHEA for 24 h.

Cell line	G-6-PDH activity on vehicle control (mU/mg)	G-6-PDH activity on DHEA treatment (mU/mg)	% Inhibition
PC-3	81.7±6.0	53.4±10.8	34.6
DU145	222.3±20.4	114.5±2.0	48.5
MDA-MB231	66.3±2.6	40.1±4.6	39.5

### Disruption to GSH metabolism does not further increase cancer cell killing

**Fig. 3:**
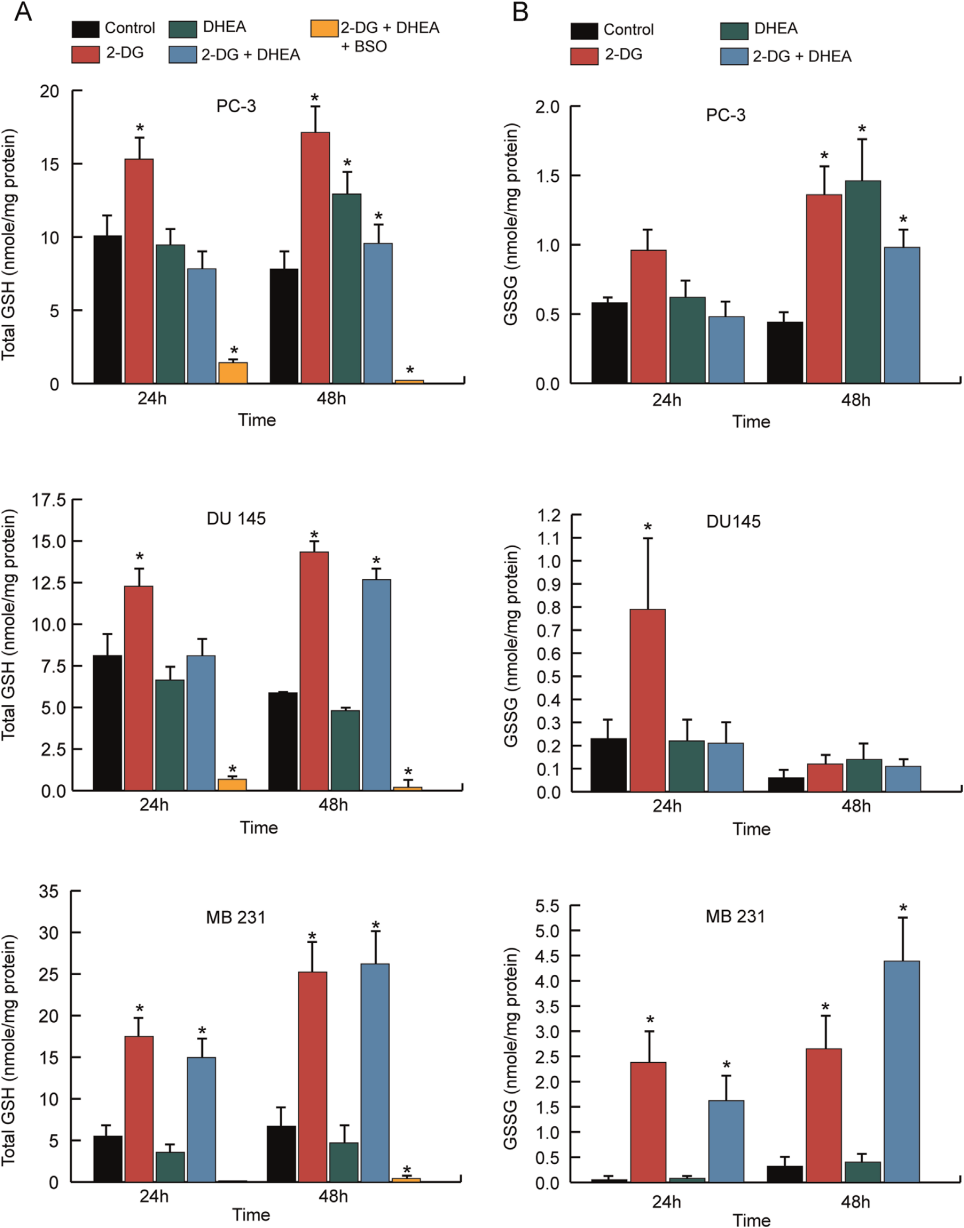
Effect of 2DG, DHEA and BSO treatment on glutathione levels in PC-3, DU145, and MDA-MB-231 cells. 1,000,000 Cells were plated into 100 mm dishes and after 24 h treated with 20 mM 2DG, 300 µM DHEA, and 1 mM BSO. At the end of 24 and 48 h total glutathione (A) and glutathione disulfide (GSSG) (B) were measured using spectrophotometric recycling assay. One way ANOVA with least significant difference (LSD) post-hoc analysis was performed resulting in *P<0.05, compared to control at the same time point.

The GSH/glutathione disulfide (GSSG) redox couple is an abundant thiol redox buffer in the cell and the ratio of GSH to GSSG is considered a good indicator of intracellular redox status as well as providing a source of reducing equivalents that protects cells from oxidative stress. GSH levels were examined in the 3 cell lines, 24 and 48 h after treatment with 2DG and DHEA (Fig. 3). Glutathione levels varied from 5 to 10 nmol/mg of protein in these cancer cell lines. Treatment with 2DG caused total GSH and GSSG levels to rise at 24 h and/or 48 h, in all 3 cell lines (Fig. 3A and B). DHEA treatment alone only increased GSH and GSSG levels in PC-3 cells at 48 h. As seen with 2DG alone, DHEA combined with 2DG treatment significantly increased total GSH and GSSG by 48 h in all 3 cell lines except for GSSG in DU145 cells. As expected following 1 mM BSO treatment, total GSH decreased to ≤10% of control (Fig. 3A). Furthermore GSSG was non-detectable in all cell lines treated with BSO (data not shown). Surprisingly, adding 1 mM BSO to the combination of 2DG and DHEA did not significantly enhance clonogenic cell killing in any of the 3 cell lines tested at 24 or 48 h (Fig. 2A–C). These results indicate that drug treatment causes disruptions to GSH metabolism. However depletion of GSH does not enhance clonogenic cell killing, suggesting that if drug treatment was mechanistically related to thiol-disruptions, total GSH content was not the critical factor determining toxicity.

### Disrupting Trx metabolism with Au potentiates 2DG and DHEA cancer cell killing

**Fig. 4:**
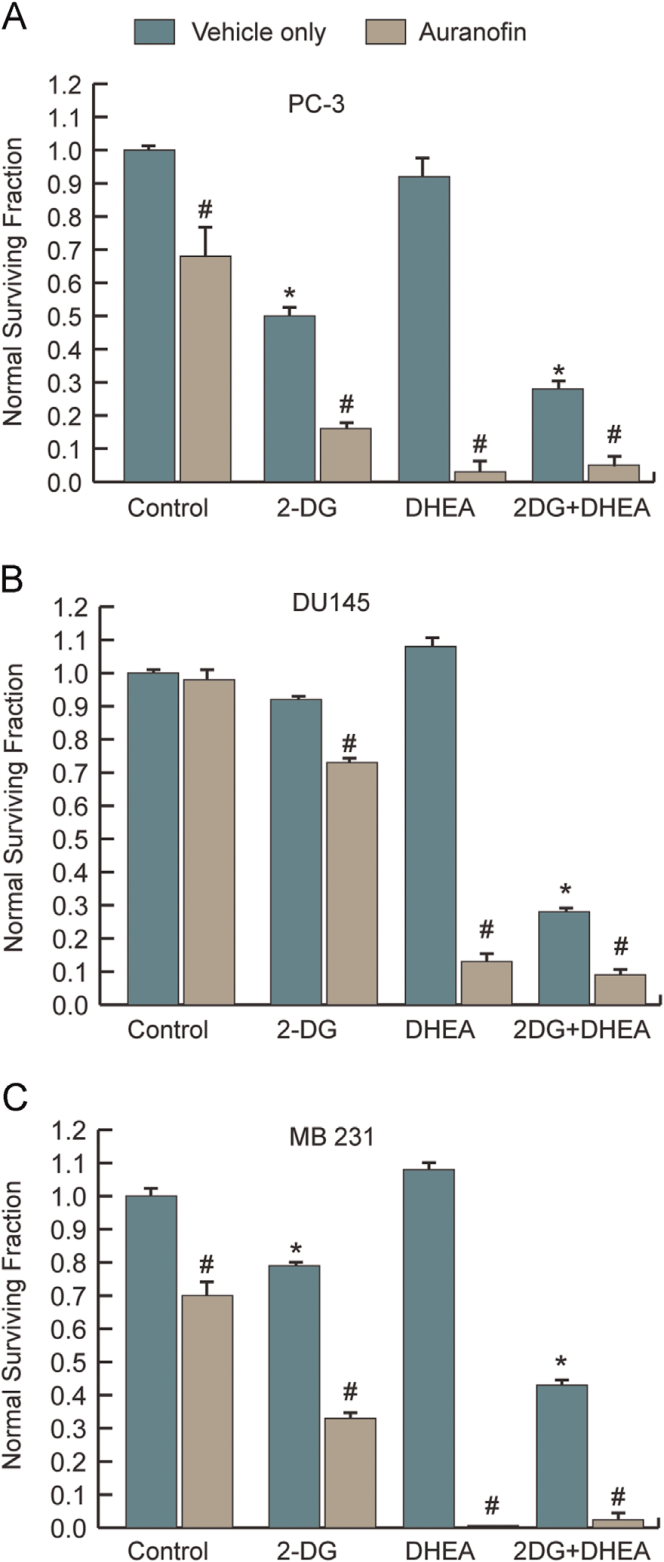
Au treatment enhances cancer cell killing from 2DG and DHEA treatment in PC-3 (A), DU145 (B), and MDA-MB-231 (C) cells. Cells were plated in 60 mm dishes and treated with 2DG and DHEA as before with the addition of 1 µM Au for 24 h after which clonogenic cell survivals assay was performed. One-way ANOVA was used with Tukey's post-hoc analysis resulted in *p<0.001 differences versus control, ^#^*p*<0.01 versus all other experimental conditions without Au.

Trx provides the reducing equivalents for the metabolism of hydroperoxides by peroxiredoxins (Prx) that plays an important role in protecting cells from oxidative stress that complements the GSH-dependent metabolism of hydroperoxides by glutathione peroxidases (GPx) (Fig. 1). When PC-3 and MB-231cells were treated with 1 µM Au for 24 h, TrxR activity was inhibited by 50–80% (*P*<0.05, *N*=3) compromising the ability of Trx and Prx metabolism to scavenge hydroperoxides in cancer cells. Treating PC-3 and MDA-MB231 cells with 1 µM Au for 24 h decreased clonogenic survival by ~30% while having no effect on DU145 cells (Fig. 4A–C). Combining Au with 2DG or with 2DG+DHEA significantly increased cytotoxicity in all 3 cell lines compared to 2DG or 2DG+DHEA treatments, respectively (Fig. 4A–C). Interestingly, Au combined with DHEA treatment also significantly increased clonogenic cell death in all three cancer cell lines compared to either drug treatment alone (Fig. 4A–C). These results support the hypothesis that Trx-mediated metabolism of hydroperoxides is relatively more important than GSH metabolism to protecting these cancer cells from oxidative stress induced by inhibition of glucose metabolism and the pentose cycle.

### NAC protects cancer cells from clonogenic cell killing mediated by DHEA and Au

**Fig. 5:**
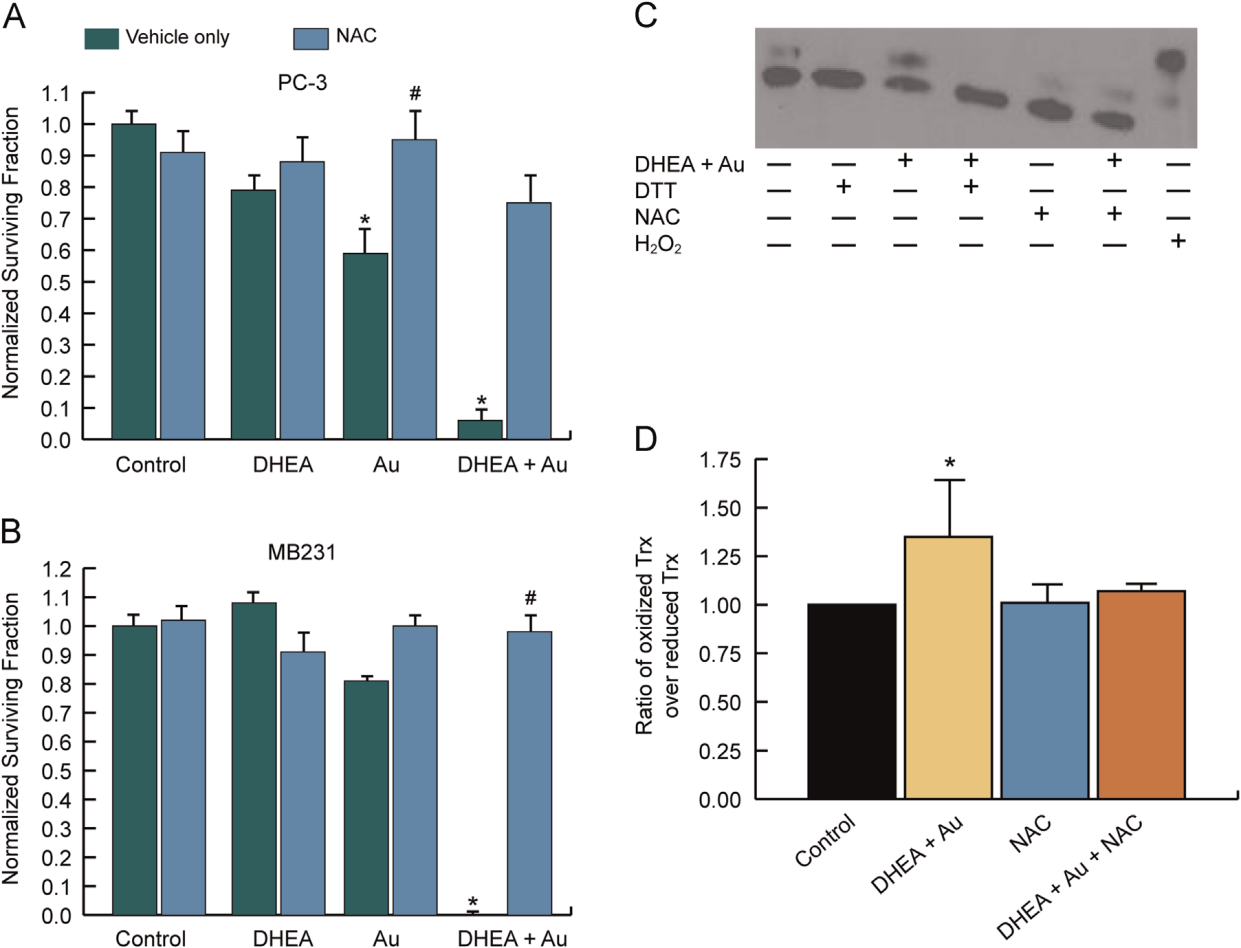
NAC rescues cancer cells from the cytotoxicity induced by combined DHEA and Au. Cells were plated and treated as above with the addition of 20 mM NAC for 24 h to select dishes followed by the clonogenic survival assay for PC-3 (A) and MB231 (B). Results were normalized to control and error bars represent mean±1SD from two different experiment treatment dishes. Each treatment dish was then plated into at least three cloning dishes each. One-way ANOVA with Tukey's post-hoc analysis resulted in *p<0.001 versus control, ^#^*p*<0.01 versus treatment without NAC. After 24 h treatment, PC-3 cells were also harvested for redox western blotting for oxidized and reduced Trx-1 as described in methods (*N*=4) (C). DTT and H_2_O_2_ were added during the final 10 min of incubation as positive and negative controls but were not included in the quantitative analysis (C). Quantification of the treatment groups included in the immuno-blot was performed using ImageJ (*N*=4) (D). One-way ANOVA followed by the LSD post-hoc analysis was used to test for statistical significance (*p<0.05).

NAC is a nonspecific thiol antioxidant that has been shown to inhibit metabolic oxidative stress [14], [35], [37]. In the current study, NAC (20 mM for 24 h) significantly inhibited clonogenic cell killing in MDA-MB-231 and PC-3 cells treated with Au or the combination of DHEA+Au (Fig. 5A and B). To further investigate the mechanisms by which NAC could act as a protective agent, redox Western blots investigating oxidation of cytoplasmic Trx-1 were performed (Fig. 5C and D). The results showed that DHEA+Au treatment caused significant Trx-1 oxidation in PC-3 cells that was inhibited by NAC (Fig. 5C and D). These data support the hypothesis that cancer cells treated with DHEA+Au have increased oxidation of intracellular thiols that is inhibited by treatment with NAC.

### Au, 2DG, and DHEA demonstrate differential cell killing in normal versus cancer breast epithelial cells

**Fig. 6:**
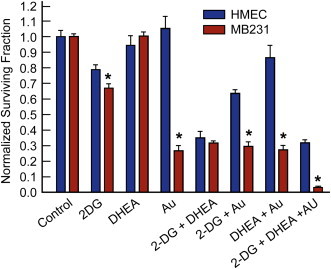
Effect of 2DG, DHEA and Au on normal breast HMEC cells versus breast cancer MDA-MB-231 cells. Cells were plated and 24 h later treated with 20 mM 2DG and 300 µM DHEA for 17–18 h. 1 µM Au was added 15 min before cells were collected for clonogenic assay. One-way ANOVA was used with Tukey's post-hoc analysis resulted in *p<0.001 when MD-MB231 cells are compared with the same treatment groups in HMEC cells.

To investigate whether these drug combinations were selectively toxic to MDA-MB-231 (human breast cancer) versus HMEC cells (normal human untransformed breast epithelial), exponentially growing cultures were exposed to combinations of 20 mM 2DG and 300 µM DHEA for 18 h followed by 1 µM Au for 15 min prior to clonogenic assay (Fig. 6). 2DG, Au, 2DG+Au, DHEA+Au and 2DG+DHEA+Au treatment groups were found to be significantly more cytotoxic to MDA-MB-231 cells, relative to HMEC cells (Fig. 6). These results demonstrate that drug treatments targeting inhibition of glycolysis and pentose cycle activity, combined with inhibition of thioredoxin metabolism are selectively cytotoxic to human breast cancer cells versus normal breast epithelial cells.

## Discussion

Cancer cells appear to have up regulated glycolytic metabolism and demonstrate some alterations aerobic respiration [1], [2], [3]. A possible consequence of this altered cancer cell metabolism is disruptions in mitochondrial electron transport chain activity which could result in increased one-electron reductions of O_2_ to form superoxide (O_2_^•−^), which can undergo dismutation reactions to become H_2_O_2_[8], [9], [40], [41]. Glucose metabolism *via* the pentose phosphate pathway leads to the regeneration of NADPH and the formation of pyruvate which have been shown to function in the cellular peroxide detoxification pathways [8], [9], [42], [43]. Both the GSH and the Trx pathways which use cysteine thiol-disulfide exchange reactions in the detoxification of H_2_O_2_ and other hydroperoxides, use NADPH for a co-factor to regenerate the reduced thiol (Fig. 1). These observations have led to the proposal that tumor cells increase their glucose utilization to form NADPH and pyruvate to compensate for the increased production of ROS (i.e., O_2_^•−^ and H_2_O_2_), which may be produced from abnormal mitochondrial electron transport chain activity [8], [9]. In support of this idea it has been demonstrated that changes in G6PDH activity, which is responsible for the regeneration of NADPH by the pentose cycle, can alter steady-state levels of intracellular ROS [25]. This suggests that inhibiting pentose cycle metabolism and peroxide detoxification pathways could preferentially kill cancer cells *via* metabolic oxidative stress. Consistent with this hypothesis, studies have shown that inhibiting glycolysis either through glucose deprivation or with 2DG preferentially induces increased cytotoxicity and oxidative stress in transformed versus non-transformed cells [8], [9], [14]. Interestingly, the clinically relevant inhibitor of glycolysis, 2DG, was found to cause less severe cancer cell cytotoxicity (relative to glucose deprivation), presumably because 2DG can only partially inhibit the pentose cycle since it is still a substrate for G6PD [9], [15].

Based on this background information, the current study assessed cancer versus normal cell toxicity associated with simultaneous inhibition of glucose metabolism in both glycolysis and the pentose cycle as well as the relative importance of GSH- versus Trx-dependent peroxide metabolic pathways in the resulting cell killing seen in human breast and prostate cancer cells. 2DG, DHEA, BSO and Au were chosen because they are well tolerated drugs in humans and they have the ability to inhibit the glucose and/or hydroperoxide metabolism, as shown in Fig. 1.

The combination of 2DG and DHEA appeared to cause at least additive cytotoxicity, as well as significant increases in total GSH and GSSG in all cancer cells tested. Surprisingly, BSO treatment depleted GSH levels but did not enhance 2DG+DHEA toxicity, suggesting that, while thiol metabolism appeared disrupted, GSH itself was not directly involved in the cytotoxic mechanism of the drug combination. It was thought that other thiol redox systems could be compensating for this stress and/or be more directly involved in the cytotoxicity induced by 2DG+DHEA treatment. Therefore, Trx metabolism was investigated for the role it might play in 2DG and DHEA induced cytotoxicity.

The antirheumatic agent, Au at a dose which inhibited TrxR activity by approximately 50–80%, resulted in a decrease in clonogenic survival of PC-3 and MB231 cells but not DU145 cells. Au treatment also enhanced the cytotoxicity of 2DG in all 3 cell lines. Most interesting was the significant decrease in clonogenic surviving fraction seen in all cancer cells when Au was combined with DHEA treatment. This increased toxicity correlated with increased Trx-1 oxidation status, suggesting that disruption to Trx metabolism was casually related to the cytotoxic mechanism of the drug combination. This hypothesis was further supported by the evidence that NAC treatment was able to protect against cytotoxicity as well as inhibit increases in the oxidation of Trx-1 seen with DHEA+Au. Furthermore, Au treatment alone or in combination with 2DG and/or DHEA caused increased clonogenic cell killing in cancer cells (MDA-MB-231) versus normal cells (HMEC). These results support the hypothesis that these drug combinations might be selectively toxic in cancer cells, providing a potential therapeutic advantage for use as an adjuvant therapy.

DHEA is an endogenous hormone produced by the adrenal glands that is present in young male athletes at approximately 0.18 µM [44] and decreases from that peak level with age. DHEA supplementation has been investigated for many age related illnesses. Doses of up to 200 mg/day have been given to large groups of subject for up to a year with very few side effects however supplementation results in at most a 25 fold increase in DHEA levels which is still lower than the 300 µM dose needed for G6PD inhibition [45]. There is active research looking for more potent, less androgenic DHEA analogs [46], [47], [48] and non-steroid G6PD inhibitors for cancer treatment [27]. Au is a gold phosphine that has been used safely in humans as an anti-rheumatoid arthritis drug for three decades and is an excellent inhibitor of both cytosolic and mitochondrial TrxR with an IC50 of 5–20 nM [33], [34]. Arthritis patients are typically given a dose of 6 mg/day orally which results in steady state serum level of 0.44–2.8 µM [49], [50] which is within the range needed for TrxR inhibition. It has been reported that Trx-1 levels are increased in several human tumors, and that higher Trx-1 levels are associated with more aggressive tumor growth [28], [29], [31]. We and others have shown that Au has antitumor activity, both *in vitro* and *in vivo* when combined with agents that increase oxidative stress [36], [51], [52], [53]. This paper is the first to show that Au combined with a G6PD inhibitor results in significant enhancement of metabolic oxidative stress in cancer cells. These results also support the hypothesis that simultaneous inhibition of glycolysis and the pentose cycle [59] is selectively cytotoxic to prostate and breast cancer cells by a pathway that can be further enhanced by inhibition of Trx metabolism. These results support the speculation that combined modality cancer therapies designed to increase metabolic oxidative stress and cancer cell killing by inhibiting the pentose cycle as well as Trx-mediated hydroperoxide metabolism may provide a useful adjuvant for the treatment of prostate and breast cancer.

## Materials and methods

### Cells and culture conditions

All the cancer cell lines were obtained from American Type Culture Collection (Manassas, MA). Human prostate cancer cell lines PC-3 and DU145, and human breast cancer cell line MDA-MB231 were maintained in RPMI-1640 medium from Mediatech, Inc. (Herndon, VA, USA) with 10% fetal bovine serum (FBS, Hyclone, Logan, Utah). Stock cultures were maintained in 5% CO_2_ and 21% O_2_ in a humidified 37 °C incubator in the absence of antibiotics. Normal non-immortalized human mammary epithelial cells (HMEC) were purchased from Lonza (Walkersville, MD) and the cells were maintained in MEBM media (Lonza), and were cultured per vendor's instructions.

### Drug treatment

2DG, DHEA, BSO, and NAC were obtained from Sigma Chemical Co. (St. Louis, MO). Au was obtained from Axxora, LLC (San Diego, CA). A final concentration of 20 mM 2DG, 300 µM DHEA, 1 mM BSO, and/or 1 µM Au were added to cells cultures in exponential growth. A stock solution of 1 M 2DG and 20 mM BSO was dissolved in phosphate-buffered saline (PBS), and 0.5 M DHEA were dissolved in DMSO. 1 mM Au was dissolved in 100% ethanol. NAC was dissolved in 1 M sodium bicarbonate (pH 7.0) immediately prior to use. 2DG, DHEA, BSO, and NAC solutions were sterile filtered prior to addition to cell cultures and the required volume added directly to the media to achieve a final concentration as mentioned above.

### Clonogenic assays

Cells were plated in 60-mm tissue culture dishes at densities to assure exponential growth for 24 h at which time 2DG, DHEA, BSO and/or Au were added as specified in the legend of each figure. Following treatment cells were trypsinized, counted and plated for clonogenic cell survival assay. Briefly, cells were diluted and plated at low densities, 200–2000 cells, in 60 mm dishes in complete media with antibiotics (gentamycin, 50 mg/L), and allowed to grow for 14 days. Surviving colonies were fixed with 70% ethanol and stained with G250 Coomassie Blue and counted (>50 cell colonies were considered survivors). The surviving fraction was defined as the number of colonies counted divided by the number of cells plated. The normalized surviving fraction was defined as surviving fraction of each dish divided by the average surviving fraction from sham treated controls with at least 3 cloning dishes per condition, repeated in at least 3 separate experiments (unless otherwise noted in legend).

### GSH/GSSG assay

Following treatment, cells were scraped and harvested in ice-cold PBS and centrifuged at 4 °C. The supernatant was discarded, and the cell pellets were frozen at −20 °C prior to biochemical analysis. Pellets were thawed and homogenized in 50 mM potassium phosphate buffer, pH 7.8, containing 1.34 mM DETAPAC. Total glutathione content was determined by the method of Anderson [54]. The yellow color of 5-thio-2-nitrobenzoic acid (TNB) generated from GSH and DTNB was detected at 412 nm. The rate at which color accumulates is proportional to the amount of total glutathione. Reduced and oxidized glutathione were distinguished by the addition of 2 µl 2-VP mixed 1:1 (v:v) with ethanol per 30 µl of sample followed by incubation for 1 h and assayed as described by Griffith [55]. Glutathione levels were normalized to the protein content using the method of Lowry et al. [56].

### G6PDH activity assay

The activity of glucose 6-phosphate dehydrogenase was measured by the method of Glock & McLean with minor modifications [57]. Immediately prior to the assay, DETAPAC buffer was added to the cell pellet and the mixture was sonicated at low power. The reaction was started by adding 12 mM 6 Phosphogluconic Acid (6PGA), 12 mM glucose-6-phosphate for the combined substrates to 2 mM NADP^+^ solution and incubating at 37 °C for 2 min. Samples were added to the reaction solutions and the spectrophotometric activity was measured for 5 min at 340 nm. The G6PDH activity is calculated by subtracting the absorbance for 6PGA alone from the combined substrate.

### Thioredoxin reductase assay

Enzymatic activity of thioredoxin reductase was determined by subtracting the time dependent increase in absorbance at 412 nm in the presence of the TrxR activity inhibitor, aurothioglucose from total activity, using the assay kit provided Sigma Chemical Co. (St. Louis, MO). One unit of activity was defined as 1 µM TNB formed/min mg protein.

### Thioredoxin redox western

Trx-1 redox Western blots were done as described [58]. Briefly, after 24 h treatment, cells were harvested by scraping directly into G-Lysis buffer containing 50 mM of sodium iodoacetate (IAA, Sigma I9148) pH 8.3, for a positive control for Trx-1 oxidation and reduction, 2 mM DTT and 2 mM H_2_O_2_ were added to some plates, incubated at 37 °C for 10 min prior to incubating with IAA. After incubating with IAA at 37 °C for 30 min, excess IAA was removed using desalting MicroSpin G-25 columns (GE Healthcare, Bio-Sciences Corp., Piscataway, NJ) [58]. Protein concentration was then determined using Bio-Rad protein assay dye reagent. 55 µg of protein were loaded onto a 15% Ready-Gel (Bio-Rad). Gels were electroblotted to a nitrocellulose membrane and probed for Trx-1 using an anti-Trx-1 primary antibody (American Diagnostica, Greenwich, CT) and horseradish peroxidase-conjugated anti-goat IgG secondary antibody, followed by chemiluminescent detection (SuperSignal West Pico, Pierce) with X-ray film. Band integrated densities were determined using imageJ software as described [37].

### Statistical analysis

Statistical analysis was done using GraphPad Prism version 4 for Windows (GraphPad Software, San Diego, CA). To determine differences between 3 or more means, one-way ANOVA with Tukey's post-hoc analysis were performed. Error bars represent the standard error of the mean. All statistical analysis was performed at the *P*<0.05 level of significance.

### Helsinki Declaration of 1975

All procedures performed are in accordance with the Helsinki Declaration of 1975.
